# ChatGPT in orthopedics: a narrative review exploring the potential of artificial intelligence in orthopedic practice

**DOI:** 10.3389/fsurg.2023.1284015

**Published:** 2023-11-01

**Authors:** Riccardo Giorgino, Mario Alessandri-Bonetti, Andrea Luca, Filippo Migliorini, Nicolò Rossi, Giuseppe M. Peretti, Laura Mangiavini

**Affiliations:** ^1^ IRCCS Istituto Ortopedico Galeazzi, Milan, Italy; ^2^ Residency Program in Orthopedics and Traumatology, University of Milan, Milan, Italy; ^3^ Department of Plastic Surgery, University of Pittsburgh Medical Center, Pittsburgh, PA, United States; ^4^ Department of Orthopaedic, Trauma, and Reconstructive Surgery, RWTH University Medical Centre, Aachen, Germany; ^5^ Department of Orthopedics and Trauma Surgery, Academic Hospital of Bolzano (SABES-ASDAA), Teaching Hospital of the Paracelsus Medical University, Bolzano, Italy; ^6^ Dipartimento di Scienze Biomediche per la Salute, Università degli Studi di Milano, Milan, Italy

**Keywords:** orthopedics, artificial intelligence, AI, ChatGPT, patient education, clinical decision-making

## Abstract

The field of orthopedics faces complex challenges requiring quick and intricate decisions, with patient education and compliance playing crucial roles in treatment outcomes. Technological advancements in artificial intelligence (AI) can potentially enhance orthopedic care. ChatGPT, a natural language processing technology developed by OpenAI, has shown promise in various sectors, including healthcare. ChatGPT can facilitate patient information exchange in orthopedics, provide clinical decision support, and improve patient communication and education. It can assist in differential diagnosis, suggest appropriate imaging modalities, and optimize treatment plans based on evidence-based guidelines. However, ChatGPT has limitations, such as insufficient expertise in specialized domains and a lack of contextual understanding. The application of ChatGPT in orthopedics is still evolving, with studies exploring its potential in clinical decision-making, patient education, workflow optimization, and scientific literature. The results indicate both the benefits and limitations of ChatGPT, emphasizing the need for caution, ethical considerations, and human oversight. Addressing training data quality, biases, data privacy, and accountability challenges is crucial for responsible implementation. While ChatGPT has the potential to transform orthopedic healthcare, further research and development are necessary to ensure its reliability, accuracy, and ethical use in patient care.

## 1. Introduction

Musculoskeletal disorders affect millions of individuals worldwide each year and orthopedic surgeons often face challenging situations requiring quick and complex decisions. Furthermore, patients' education and compliance in orthopedics are essential in improving treatment outcomes and active participation in recovery (1).

Over the years, technological advancements have significantly influenced the practice of orthopedics, with the integration of artificial intelligence (AI) systems showing great potential in improving patient care and outcomes. In fact, this new imposing reality is developing exponentially in the healthcare sector, especially due to the improvement in computing power, the increase in health data, and the ability to access large sets of exploitable data (2). There are numerous stages of patient management where AI could play a useful role, ranging from the diagnostic to the therapeutic aspect. Among the various AI-based systems, ChatGPT, a language natural processing (LNP) technology developed by OpenAI (San Francisco, CA), was launched in November 2022.

ChatGPT is one of the LNP models based on the transformer architecture and trained on a vast corpus of textual data, enabling it to generate human-like responses to user questions in an interactive way. Its ability to understand and generate contextually relevant and coherent responses has led to its exploration and application in various sectors, including healthcare. In the field of orthopedics, this AI-based tool can provide clinical contributions to the complex decision-making process by facilitating information exchange with patients and providing accessible and accurate information to both healthcare professionals and patients themselves. The trends AI research following the launch of ChatGPT have recently been analyzed with the aim of identifying key developments and future directions. Alessandri-Bonetti et al. conducted a bibliometric analysis of the literature in the first 7 months since the introduction of ChatGPT until July 1st, 2023, collecting 724 articles (3). A significant increase in publications exploring ChatGPT use across various medical disciplines has been observed, especially in the medical field, suggesting a growing relevance of ChatGPT in the healthcare sector. Interestingly, a decrease in studies focused on ethical considerations has been noted, indicating a shift in research focus. The results highlight the increasing integration of ChatGPT in various medical disciplines, underscoring its expanding role in healthcare.

Among all areas of medicine, orthopedics deserves particular attention. Orthopedic conditions encompass a wide range of pathologies, including fractures, joint disorders, spinal deformities, and sports injuries. ChatGPT has the potential to serve as a clinical decision-support tool by providing clinicians with relevant information based on patient symptoms, medical history, and radiological findings. Its features can be helpful in differential diagnosis and suggest diagnostic tests or appropriate imaging modalities for further evaluation. Therapeutic recommendations in orthopedics are often based on evidence-based guidelines and clinical experience. AI technologies can optimize this process by assisting clinicians in synthesizing a vast amount of medical literature and providing updated therapeutic recommendations based on the specific characteristics of the patient and their condition. This can contribute to optimizing treatment plans, promoting adherence to evidence-based practices, and reducing variability in clinical decisions. Furthermore, ChatGPT could play a fundamental role in patient communication and education. Orthopedic conditions can often be complex, and patients often have numerous questions and concerns about their diagnosis, treatment options, and expected outcomes. ChatGPT can provide patients with reliable and understandable information, addressing their questions and alleviating their anxieties. Also, patients might enhance their knowledge and preparedness prior to surgeon's consult, potentially resulting not only in patient's readiness but also time saving for the physician. This can lead to improved patient satisfaction, interaction, and adherence to treatment plans. While this perspective could open up new opportunities for patients, it is likely dangerous to envision the use of ChatGPT as a substitute for the physical examination by a medical professional or specialist consultation.

Finally, ChatGPT can be a valuable tool for literature review and research in orthopedics, which is continuously evolving, with new studies and publications being released regularly. Keeping up with the latest evidence can be a challenge for clinicians and researchers. ChatGPT can assist in conducting literature searches, summarizing research articles, and identifying key findings, thus facilitating evidence-based practice and promoting knowledge translation. The significant impact of Artificial Intelligence in writing or assisting researchers has led several international scientific journals to require the declaration of whether AI software was used in writing an article. Indeed, despite the numerous potential advantages, it is essential to ensure scientific integrity and ethics in AI-assisted research and writing. Simultaneously, transparency regarding the use of AI in documents is a mandatory step towards genuine scientific responsibility.

Protecting this and many other aspects must be mandatory in approaching this pivotal shift in the medical and orthopedic world. The aim of this review is to provide a comprehensive overview of the use of ChatGPT in orthopedics, highlighting the pros and cons of each application. By synthesizing the available evidence, we hope to shed light on the strengths, limitations, and future implications of ChatGPT in enhancing patient care, clinical decision-making, and workflow optimization. The findings of this review will inform healthcare professionals, researchers, and policymakers about the current state of knowledge in this field and provide guidance for future research and implementation of ChatGPT in orthopedic practice.

## 2. Materials and methods

Studies were searched on PubMed database using the keywords “ChatGPT” OR “language natural processing” AND “Orthopaedics”. Last search was conducted on July 1st, 2023. Only studies describing the application of ChatGPT in orthopedics were included in the review. Studies involving the use of ChatGPT in orthopedic settings, such as clinical practice, patient education, decision support, and remote monitoring, will be considered. Exclusion criteria will include studies not relevant to orthopedics, non-English articles, and studies with inadequate information on the use of ChatGPT.

Two independent reviewers (R.G. and A.L.) performed the study selection, data extraction, and quality assessment. Any discrepancies will be resolved through consensus or consultation with a third reviewer (M.A.B.). The extracted data will include study characteristics, study design, and key findings.

## 3. Results

A diverse range of studies on the use of ChatGPT in orthopedics was observed. The results are presented in a narrative synthesis, organized according to the different domains of orthopedic practice in which ChatGPT has been utilized. The main topics in which ChatGPT was tested were clinical decision-making, patient education, and workflow optimization. Table 1 provides key study characteristics.

**Table 1:** Key study caractreristics.

Author	Year, month	Study design	Key findings
Poduval et al.	2020, January	Narrative review	Discussion of key concepts in the field of artificial intelligence and directions for its application in orthopaedics.
Cheng et al.	2023, April	Letter to editor	Identification of the major roles of GPT-4 including scientific research, disease diagnosis, treatment options, preoperative planning, intraoperative support, and postoperative rehabilitation for arthroplasty doctors.
Hernigou et al.	2023, August	Editorial	Exploration of the potential of AI in orthopedic surgery, encompassing its ability to analyze vast datasets and generate valuable insights. This includes its role in diagnosis, preoperative planning, clinical decision support through predictive analysis, and personalized treatment planning.
Karnuta et al.	2023, June	Original article	Hypothesis of a genuine revolution in orthopedic practice, particularly in areas like personalized patient care, image analysis, and surgical decision-making. Suggestion of providing a specialized training and exposure AI systems to orthopedic texts and manuscripts to enhance their performance.
Dubin et al.	2023, April	Original article	Comparison between the appropriateness and reliability of ChatGPT and Google web search as resources for patients seeking health information online.
Cuthbert et al.	2023, September	Original article	Evaluation of ChatGPT in passing Section 1 of the Fellowship of the Royal College of Surgeons (FRCS) examination in Traumatology and Orthopedic Surgery.
Alessandri-Bonetti et al.	2023, July	Letter to editor	Assessment of ChatGPT's medical knowledge in comparison to graduate medical doctors in Italy through its performance in the Italian Residency Admission National Exam.
Bi et al.	2023, April	Original article	Discussion of the accuracy of the generated content and the necessity for quality control and fact-checking.
Olliver et al.	2023, March	Editorial	Discussion about the issues of plagiarism and false content in scientific literature.
Kunze et al.	2023, June	Editorial	Critical evaluation of transparency, responsibility, and thorough evaluation of AI systems, aiming to improve their reliability and the quality of the generated results.
Parsa et al.	2023, April	Editorial	Discussion of data privacy concerns, quality control, biases in training data, and the challenge of attributing authorship in AI-generated content.

## 4. Discussion

### 4.1. Exploring the diverse applications of ChatGPT in orthopedics: from diagnosis to treatment planning

The use of ChatGPT in the field of orthopedics has started to be explored in the scientific literature, with numerous articles discussing its potential. As reported in the work of Poduval et al. (4), it is now essential to understand and embrace robotics and AI, along with traditional clinical skills, in modern orthopedic practice. Since AI has the potential to be a positive and disruptive force in orthopedic surgery, orthopedic surgeons must accept and explore its possibilities. Indeed, advantageous prospects can be found in improving diagnostic accuracy, optimizing surgical planning, providing effective intraoperative assistance, and personalizing treatments. At the same time, the potential disruptive force of this technology must be monitored in areas such as data security, the need for continuous medical supervision, and the maintenance of medical ethics and integrity.

According to Cheng et al. (5), the main roles of ChatGPT can be found in scientific research, disease diagnosis, treatment options, preoperative planning, intraoperative support, and postoperative rehabilitation.The incredible potential of AI in orthopedic surgery is further discussed in the paper by Hernigou (6). According to the authors, the unique characteristic of AI that is well-suited to this medical field is its ability to analyze large amounts of data and generate useful information. With this feature, AI can not only assist in diagnosis, preoperative planning, or intraoperative guidance but also provide clinical decision support based on predictive analysis and personalized treatment plans.

Karnuta et al. (7) even compare the transformative potential of AI technology to historical advancements such as the introduction of metallic instruments and the Industrial Revolution. In this article as well, the authors hypothesize a real revolution in orthopedic practice in areas such as personalized patient care, image analysis, and surgical decision-making. To overcome the current limitations of ChatGPT in synthesizing complex orthopedic knowledge and answering intricate questions, the authors suggest that specialized training and exposure to orthopedic texts and manuscripts could enable AI systems to achieve higher performance levels and even surpass orthopedic exams. Although clinical applications are still lacking, and the AI technology still appears weak in real-life complex scenarios, according to the available reports, it is foreseeable that ChatGPT potential or future AI models will dramatically change orthopedics practice.

### 4.2. Empowering patients with AI: assessing the role of ChatGPT in providing reliable health information

In today's world, where patients have access to a vast amount of data (often not accurate and up-to-date), a crucial role could be played by this AI-based tool in patient information. In the paper by Dubin et al. (8), a comparison is made between the appropriateness and reliability of ChatGPT and Google web search as resources for patients seeking health information online. The study compares frequently asked questions (FAQs) related to total knee arthroplasty (TKA) and total hip arthroplasty (THA) obtained from both sources. Only 25% of the questions were similar when performing a Google web search and a search of ChatGPT for all search terms, with 13/20 Google results from commercial sites and 15/20 ChatGPT results from government sites. 11/20 numerical questions had different responses. ChatGPT provided heterogeneous questions and responses. In conclusion, it is not yet a reliable source of information for patients. More research is needed to determine its accuracy and reliability. Until then, patients should consult with a healthcare professional for medical questions or concerns.

### 4.3. Challenging ChatGPT

ChatGPT has been put to the test in various fields of medicine, and some have even attempted to “challenge” the AI-based ChatGPT model in the field of orthopedics, comparing it to human knowledge. The interesting work by Cuthbert (9) aimed to evaluate whether ChatGPT could pass Section 1 of the Fellowship of the Royal College of Surgeons (FRCS) examination in Traumatology and Orthopedic Surgery. The results demonstrated that ChatGPT achieved only 35.8%, significantly lower than the passing rate of FRCS and the average score obtained by human candidates at all levels of training. The main shortcomings of ChatGPT were identified in its inability to exercise higher-order judgment and the multilogical thinking required to pass the examination. These limitations should be recognized and publicized to ensure that clinicians are aware of them. The results of this study also underline the importance of critically assessing the reliability and limitations of artificial intelligence systems in the context of real-life complex scenarios. While ChatGPT has shown promise in generating contextually relevant text, its performance in a highly specialized and technical domain like orthopedic surgery has been insufficient. This suggests that AI models like ChatGPT may not necessarily possess the necessary expertise and clinical reasoning skills required for complex medical decisions. Additionally, the study revealed that ChatGPT failed to recognize its own limitations, providing incorrect explanations for questions it answered incorrectly. This represents a significant and dangerous limitation of this tool. Clinicians and educators should be cautious about relying solely on artificial intelligence systems for assessments or decisions without understanding their limitations and ensuring adequate human oversight. Adapting the training data and refining the model with specialized orthopedic knowledge could enhance its performance in this domain. Furthermore, efforts should be made to address the lack of contextual understanding exhibited by ChatGPT, as this is a crucial aspect of clinical decision-making. A recent study aimed to evaluate ChatGPT's performance in the Italian Residency Admission National Exam to assess its level of medical knowledge compared to graduate medical doctors in Italy (10). In June 2023, ChatGPT3 was employed to undertake this exam, which consists of a computer-based multiple-choice test comprising 140 questions, taken annually by all Italian medical graduates. The exam evaluates basic medical science knowledge and its application. ChatGPT's performance was compared with that of 15,869 medical graduates, revealing that ChatGPT answered 122 out of 140 questions correctly. The score ranked in the 98.8th percentile among the 15,869 medical graduates. Among the 18 incorrect answers, 10 related to direct questions about basic medical science knowledge, while 8 concerned applied clinical knowledge and reasoning through case presentations. Errors were logical (2 incorrect answers, ChatGPT motivated correctly the answer, but provided the wrong multiple-choice answer) and informational in nature (16 incorrect answers, ChatGPT provided incorrect answer and reasoning). Interestingly, all explanations for correct answers were deemed “appropriate.” Comparing with national statistics regarding the minimum score required to access each specialty, ChatGPT's performance demonstrated it would have qualified the candidate for any specialty. Thus, ChatGPT displayed competence in basic medical science knowledge and applied clinical knowledge. Further research should evaluate ChatGPT's impact and reliability in clinical practice.

### 4.4. ChatGPT in scientific literature: opportunities, challenges, and the imperative of ethical standards

There is another important aspect where ChatGPT is gaining traction, namely the field of scientific literature. The potential uses can vary widely, ranging from grammar correction and proofreading to planning the highlights of scientific articles. According to Bi et al. (11), the ability of ChatGPT to generate manuscript drafts and its potential to streamline the writing process should be acknowledged. However, concerns are raised about the accuracy of the generated content and the need for quality control and fact-checking. Ollivier et al. (12) discuss the problem of plagiarism and false content in scientific literature. We agree with the points raised by the authors, emphasizing the importance of maintaining high ethical standards and accuracy in scientific research. While large language models like ChatGPT have the potential to assist in text generation and information synthesis, it is essential to critically evaluate their results for scientific validity. The authors propose measures such as data sharing, improved training and education, and the development of technologies and tools to detect plagiarism and misconduct. The need to verify and corroborate the information generated by AI models, as well as the importance of ethical standards, transparency, and reliability in scientific research and publication, remain pressing. The role of human evaluation and critical thinking is still indispensable for the effective and responsible use of AI-generated content.

### 4.5. Cautions and recommendations

Given the aforementioned points, we feel obligated to provide cautions and recommendations for the interpretation of data derived from ChatGPT, as already shared by many authors of the aforementioned studies. New horizons and challenges such as data privacy, security, validation, and ethical considerations arise when ensuring responsible implementation of AI in orthopedic surgery. The responsible use of this tool must be based on an awareness of its limitations and biases. Foremost among them is the dangerous concept of AI hallucination (6). This phenomenon involves the possibility of generating incorrect responses but still providing confident and plausible-sounding explanations. The authors cite the following example: when asked to generate a report on an event after its last update, the chatbot falsely discusses the announcement but later admits that it has no information about this communication because it lacks temporal data availability. AI can rely on machine learning algorithms trained on extensive datasets to assess source credibility through reputation analysis and source consistency, aiming to identify potential patterns of misinformation or the spread of false information. Another tool at its disposal is spoken natural language analysis, which, through semantic and syntactic examination, can help recognize inaccurate or misleading information. Despite having these resources, AI hallucinations can be extremely perilous when critical analysis of obtained information is not conducted. Therefore, careful scrutiny is needed to avoid the inadvertent distribution of misleading or inaccurate medical knowledge.

Another aspect that deserves caution is the potential risk of bias in ChatGPT's responses. The generated answers could be influenced by the training data, which may reflect biases or trends in the original texts that are not necessarily accurate or up-to-date. This could manifest as formulating biased or unrepresentative recommendations or diagnoses in orthopedics. Therefore, we emphasize the importance of conducting a critical assessment of the responses and considering possible measures to mitigate any bias. Another challenge may lie in the demand for Structured Content Generation by ChatGPT. In the field of orthopedics, this could translate into the creation of orthopedic medical reports, which require strict formatting and organization of information. In this case, we also recommend a careful manual review of the generated documents to ensure the proper structuring of data.

Other peculiar elements that deserve attention are described in the article by Karnuta et al. (7), such as the “garbage in, garbage out” principle, emphasizing the importance of ensuring high-quality and unbiased data as input for AI systems to avoid perpetuating biases and misinformation. The same article also discusses the responsibility and obligation to ensure robust safety mechanisms and clear roles for stakeholders in the event of system malfunctions and harm to patients (7). These models may not possess in-depth domain-specific knowledge and may lack the ability to apply higher-order judgment and reasoning, especially in complex medical contexts (13). Aspects such as transparency, responsibility, and thorough evaluation of AI systems need to be sought and improved to ensure the reliability and quality of the generated results (13). Lastly, there are other elements worth mentioning, such as data privacy, quality control, biases in training data, and the challenge of authors' attribution (5, 14). Therefore, careful regulation and ethical use of tools like ChatGPT in orthopedics and medicine seem necessary.

Finally, we recommend caution in managing multiple tasks simultaneously. Although ChatGPT can handle a wide range of tasks, this could pose limitations. In the field of orthopedics, a physician may have to address multiple questions simultaneously in a single interaction with ChatGPT. This may necessitate greater care in formulating questions and interpreting responses to ensure no confusion and thus provide accurate answers.

With these considerations, physicians should actively shape the trajectory of AI, providing feedback to regulatory bodies and developers, promoting dialogue, and ensuring a thorough examination of the implications of AI implementation in clinical practice (7).

### 4.6. Strengths and limitations

To the best of our knowledge, this is the first review of ChatGPT in the orthopedic field. This paper provides a comprehensive overview of the use of ChatGPT in orthopedics, covering various aspects such as clinical decision-making, patient education, workflow optimization, and scientific literature. The present study presents both the potential benefits and limitations of using ChatGPT, highlighting the need for caution, ethical considerations, and human oversight.

This study also presents several limitations. First of all, it is a narrative review. So, although the review mentions the use of independent reviewers and quality assessment tools, it does not follow a standard systematic review methodology, such as a predefined protocol or PRISMA guidelines. Secondly, there is a lack of critical appraisal. This narrative review does not provide an evaluation of the quality or risk of bias of individual studies. A critical appraisal of the included studies would allow readers to assess the strength of the evidence presented. In conclusion, while this narrative review provides a comprehensive overview of the potential applications of ChatGPT in orthopedics and highlights the need for caution and ethical considerations, its limitations as a non-systematic review and lack of critical appraisal of included studies should be considered when interpreting the findings. 

## 5. Conclusions

The integration of AI technologies, including ChatGPT, holds tremendous promise for transforming orthopedic healthcare. Although the potential applications of ChatGPT in orthopedics are promising, several challenges and considerations need to be addressed. The reliability and accuracy of the responses generated by ChatGPT depend on the quality of the training data and algorithms used. It is essential to ensure that the language model is trained on diverse and high-quality orthopedic data to minimize the risk of bias and incorrect recommendations. Furthermore, ethical and legal aspects of AI use in healthcare, such as data privacy, security, and accountability, must be carefully addressed to ensure patient confidentiality and trust.

Addressing the challenges and considerations associated with its use is crucial to ensure the reliability, accuracy, and ethical implementation of this technology. Ongoing research and development in this field will pave the way for the integration of ChatGPT and other artificial intelligence systems in orthopedics, benefiting both patients and healthcare providers.
